# Integrative single-cell transcriptomic investigation unveils long non-coding RNAs associated with localized cellular inflammation in psoriasis

**DOI:** 10.3389/fimmu.2023.1265517

**Published:** 2023-09-26

**Authors:** Yuge Gao, Mengxue Na, Xinyu Yao, Chao Li, Li Li, Guangyu Yang, Yuzhen Li, Yizhou Hu

**Affiliations:** ^1^ Department of Dermatology, The Second Affiliated Hospital of Harbin Medical University, Harbin, China; ^2^ Department of Dermatology, Peking University First Hospital, Beijing, China; ^3^ Department of Oncology, The Second Affiliated Hospital of Harbin Medical University, Harbin, China; ^4^ Department of Medical Biochemistry and Biophysics, Karolinska Institute, Stockholm, Sweden

**Keywords:** psoriasis, lncRNA, single cell, skin, transcriptomic atlas, localized inflammation

## Abstract

Psoriasis is a complex, chronic autoimmune disorder predominantly affecting the skin. Accumulating evidence underscores the critical role of localized cellular inflammation in the development and persistence of psoriatic skin lesions, involving cell types such as keratinocytes, mesenchymal cells, and Schwann cells. However, the underlying mechanisms remain largely unexplored. Long non-coding RNAs (lncRNAs), known to regulate gene expression across various cellular processes, have been particularly implicated in immune regulation. We utilized our neural-network learning pipeline to integrate 106,675 cells from healthy human skin and 79,887 cells from psoriatic human skin. This formed the most extensive cell transcriptomic atlas of human psoriatic skin to date. The robustness of our reclassified cell-types, representing full-layer zonation in human skin, was affirmed through neural-network learning-based cross-validation. We then developed a publicly available website to present this integrated dataset. We carried out analysis for differentially expressed lncRNAs, co-regulated gene patterns, and GO-bioprocess enrichment, enabling us to pinpoint lncRNAs that modulate localized cellular inflammation in psoriasis at the single-cell level. Subsequent experimental validation with skin cell lines and primary cells from psoriatic skin confirmed these lncRNAs’ functional role in localized cellular inflammation. Our study provides a comprehensive cell transcriptomic atlas of full-layer human skin in both healthy and psoriatic conditions, unveiling a new regulatory mechanism that governs localized cellular inflammation in psoriasis and highlights the therapeutic potential of lncRNAs in this disease’s management.

## Introduction

Psoriasis is a genetically related, immune-mediated chronic inflammatory systemic disease characterized by skin manifestations, with a prevalence rate of approximately 2% to 3% worldwide, leading a significant burden of disease (1). Psoriasis can cause skin redness, swelling, scales, and other systems such as joints, blood vessels, and intestines, and relate to the occurrence of metabolic diseases such as diabetes.

An increasing number of cell subtypes have been implied to contribute to psoriasis pathogenesis. These cell subtypes interact and form a complicated regulatory network, impacting one another in numerous ways. Key genetic loci within skin-resident cell types can directly affect non-immune cell immune regulation, resulting in localized skin cell inflammation (2). This persistent inflammatory state, either directly or indirectly, contributes to psoriasis onset. Therefore, the localized inflammation in the skin is a distinct hallmark of psoriasis. Despite substantial strides in understanding the underlying mechanisms of psoriasis, effective treatments specifically addressing the localized inflammation seen in psoriatic skin are yet to be discovered. Notably, the epigenetic regulatory mechanisms governing inflammation within resident skin cells remain largely elusive.

Non-coding RNAs (ncRNAs), a class of RNA molecules that do not encode proteins, have emerged as critical regulators of gene expression, and aberrant ncRNA expression has been implicated in various diseases, including psoriasis (3). Among ncRNAs, long non-coding RNAs (lncRNAs) have garnered considerable attention for their diverse functions in regulating gene expression, ranging from transcriptional and post-transcriptional regulation to epigenetic modulation. Recent studies have shown that lncRNAs can modulate various cellular processes involved in inflammation and immune regulation, suggesting a potential role for lncRNAs in psoriasis pathogenesis (4, 5).

The advent of single-cell transcriptomics has revolutionized the field of genomics, enabling the study of gene expression at the resolution of individual cells. It can define cell subpopulations with potential therapeutic targets and characterize the specific responses of cell subpopulations to drugs or other stimuli (6). In the context of psoriasis, single-cell transcriptomic (scRNAseq) datasets generated from different research groups have revealed the cellular diversity of psoriasis lesions and identified novel cell populations involved in the pathogenesis of psoriasis (2, 7, 8). scRNAseq can discover new cell subpopulations and evaluate organ- or tissue-specific transcriptomic features of keratinocytes (KCs), fibroblasts, endothelial cells, and immune cells that are involved in inflammation or infiltration, elucidating the functional heterogeneity of cells in psoriasis. It is also used to analyze cell distribution and cell-to-cell communication, providing new clues to the complex interactions between components involved in disease response (9). However, the data batch exists across these datasets, obstacle the skin cell classification and the accuracy of exploring the underlying pathogenesis of psoriatic skin.

In this study, we employed our neural-network learning pipeline to integrate 106,675 cells from healthy human skin and 79,887 cells from psoriatic human skin. This integration resulted in the most comprehensive cell transcriptomic atlas of human psoriatic skin so far. Cross-validation, grounded on neural-network learning, affirmed the validity of our reclassified cell-types, portraying full-layer zonation in human skin. We also launched a publicly accessible website featuring this consolidated dataset (https://yz-studio.shinyapps.io/psoriaticskincellatlas2/). Furthermore, this study pioneers the use of single-cell transcriptomics of entire skin tissue to pinpoint lncRNAs that modulate localized inflammation in psoriasis at the cellular level. We profiled transcriptomes of individual cells isolated from psoriatic lesions and healthy skin, identifying novel differentially expressed lncRNAs involved in localized inflammation. These lncRNAs, previously not associated with psoriasis, constitute promising candidates for future research and therapeutic development. Our study furnishes novel insights into the regulatory mechanisms at the heart of psoriasis pathogenesis, and underscores the power of single-cell transcriptomics in discerning disease-relevant cell populations and molecular targets.

## Materials and methods

### Single-cell data analysis

The single-cell data analysis was described in our previous study (10, 11), including neural network learning and visualization, differential gene expression in each cell-type, GO enrichment. Most analysis tools have been integrated in the toolkit scCAMEL with tutorials online: https://sccamel.readthedocs.io/. The website representing our integrated cell atlas were constructed by using Shinny apps. To strike a balance between accuracy and efficient website navigation, we downsampled the cell number to maximum 300 for each cell type (12), under each condition, and for each donor. This resulted in a total of 49,237 cells available for website visualization.

### Improved SWAPLINE integration and projection

We enhanced the SWAPLINE package by augmenting its adaptability during dataset integration and projection, thus generating an updated version, as detailed in our recent publication (10, 11). Briefly, we utilized an interpretable neural network for training each dataset and predicting all other datasets. The probabilistic scores derived from both trained and predicted datasets form the latent space for subsequent analysis. Using this approach, we successfully integrated a healthy skin reference dataset and reclassify the cell types to reflect the full-layer skin zonation. The improved SWAPLINE and the integration case study will be published separately.

For projecting human healthy and psoriatic skin datasets in this study, we computed the probabilistic score for each cell within each query dataset in the trained reference datasets, building the latent space for label transfer analysis in nearest neighbor model. At the same time, gene expression normalization and denoising were carried out using an interpretable learning nearest neighbor model. Feature weights for each reference cell type were estimated using the DeepLift algorithm in the Captum package for PyTorch. The gene expression for each cell, either learned or predicted in a trained reference dataset, was inferred through matrix multiplication of the feature weights and cell-type probabilistic scores. In the end, the gene-cell expression matrix was computed by averaging non-empty values across all datasets. Therefore, with a ready-built reference skin cell transcriptomic atlas, we successfully project the healthy and psoriatic skin cells from three public datasets, recovered the expression of most protein-coding genes and lncRNAs (2, 8, 9).

### Sample collection and ethics approval

This experiment recruited volunteers with diagnosed psoriasis and healthy controls, all of whom signed an informed consent form and underwent ethical review declaration. Typical lesion sites on the trunk were selected, and after disinfection, full-thickness skin samples were surgically excised and excess subcutaneous tissue and fat were removed. Cell digestion solution was prepared in RPMI 1640 10% FBS medium with collagenase IV (VETEC, USA) 200 U/ml and DNase I (Solarbio, CHN) 200 µg/ml at appropriate concentrations. The tissue was minced and digested overnight in a 37°C, 5% CO2 incubator. The resulting single-cell suspension was obtained after centrifugation and filtration and was used for flow cytometry cell sorting.

### Flow cytometry

For the flow cytometric analysis sorting, skin cells were sorting according to Propidium Iodide (Biosharp, China). The PI-negative cells were immunolabeled with *IL-20RB* Polyclonal antibody (Proteintech, USA), then immunolabeled with Goat Anti Rabbit IgG(H&L)-Alexa Fluor 488 and PE anti-human *CD140b* (*PDGFRB*) Antibody (Biolegend, USA). *IL20RB*-positive and *PDGFRB*-positive cells were collected on Beckman MoFloXDP for RT-qPCR analysis of gene expression levels, the result was analyzed by FlowJo.

For flow cytometry, live DCs were immunolabeled with APC-CY7-livedead (Thermo Fisher Scientific, Massachusetts, USA), Brilliant Violet 605™ anti-mouse *CD11C* (BioLegend, CA), PE anti-mouse *CD80* (Thermo Fisher Scientific), FITC anti-mouse *CD86* (Thermo Fisher Scientific) at 4°C for 30 min. All cells were detected on Beckman Cytoflex LX and the result was analyzed by FlowJo.

### RT-qPCR

Dispersed cell suspensions or cell samples were extracted with TRIzol (Invitrogen, Australia) to obtain total RNA. All-in-one First Strand cDNA Synthesis Kit (Seven, CHN) was used to prepare the DNA library. The reaction was terminated by incubating at 85°C for 5 seconds. Quantitative real-time PCR was performed using the 2× SYBR Green kit (Seven, CHN) on a fluorescence quantitative PCR detection system (Hangzhou Bori, CHN). The reaction was performed according to the manufacturer’s instructions. Relative gene expression was calculated using the 2-ΔΔCT method. All experiments were repeated three times.

Primer sequence as follows: *SNHG9* human F: GACTGCAGACCCCTAACCTT; R: ACCCGCATGCAGTGAGTTA. *CALML3-AS1* human F: TGCAGTGTCACTCTGGAAGC; R: CACTGTCTCAGGCCAGGTTT. *CARMN* human F:AGGAGAGCAACGGCTGTAAC; R:TCTCTGACATCAGCATGGCG. *GAPDH* human F: CATGTTCGTCATGGGTGTGAA; R: GGCATGGACTGTGGTCATGAG. *CXCL8* human F: TTTTGCCAAGGAGTGCTAAAGA; *CXCL8* human R: AACCCTCTGCACCCAGTTTTC. *CCL20* human F: TGCTGTACCAAGAGTTTGCTC; *CCL20* human R: CGCACACAGACAACTTTTTCTTT. *IL-17B* human F: AGCCCCAAAAGCAAGAGGAA; *IL-17B* human R: TGCGGGCATACGGTTTCATC. *IL-1B* human F: ATGATGGCTTATTACAGTGGCAA; *IL-1B* human R: GTCGGAGATTCGTAGCTGGA. *IL6* human F: ACTCACCTCTTCAGAACGAATTG; *IL6* human R: CCATCTTTGGAAGGTTCAGGTTG.

### Cell culture and RNA interference

HACAT cells were cultured in DMEM medium containing 10% fetal bovine serum. HSF cells were cultured in F12 medium containing 10% fetal bovine serum. siRNAs were used to silence *SNHG9/CALML3-AS1* in the HACAT cell line and *CARMN* in the HSF, transfected with Lipo8000 transfection reagents (Beyotime,CHN) following the product manual, with cells treated with blank siRNA serving as negative controls. Cells were stimulated with recombinant human TNF-α protein (Abcam) 50ng/ml to simulate localized inflammation.

siRNA sequences targeting the lncRNA as follow: *CALML3-AS1-Homo-2405*: GGUGUUCCUCGCAUGACUUTT; AAGUCAUGCGAGGAACACCTT. *CARMN-Homo-899*: CCUGUGCUCUGUGACAAUATT; UAUUGUCACAGAGCACAGGTT. *SNHG9-Homo-78*: CCCGAAGAGUGGCUAUAAATT; UUUAUAGCCACUCUUCGGGTT.

### Co-culture of skin resident cells and dendritic cells

pDCs purchased from ATCC, and were cultured in 1640 medium containing 10% fetal bovine serum, 1% penicillin/streptomycin, 10ng/ml recombinant human IL-4 protein, and 20ng/ml granulocyte-macrophage colony-stimulating factor (GM-CSF) under 37°C and 5% CO2 for 7 days. After washing and collecting the cells, they were mixed with dendritic cells and cultured overnight in 1640 medium containing 10% fetal bovine serum under 37°C and 5% CO2. Dendritic cells were labeled with CD11C, and CD80 and CD86 were used as activation markers. Flow cytometry was used to detect the cells, and the proportion of CD80 and CD86 positive cells in CD11C positive dendritic cells was calculated to evaluate the degree of DCs activation by skin-resident cells in different groups.

### Statistical analysis

Statistical analysis was performed by GraphPad Prism 9 software. All the data values were presented as means ± SEM. The statistical significance was assessed by Student’s unpaired two taied t test for the two-group comparison. Each experiment was repeated at least three times. *P* < 0.05 was considered statistically significant (∗*P* < 0.05, ∗∗*P* < 0.01, ∗∗∗*P* < 0.001, ∗∗∗∗*P* < 0.0001).

## Results

### Neural-network learning based integrative analysis constructs the cell transcriptomic atlas of psoriatic skin

By employing our recently developed interpretable neural-network analysis toolkit, we successfully integrated 106,675 cells from healthy human skin and 79,887 cells from psoriatic human skin across three public datasets (2, 7, 8). Through this, we construct the largest transcriptomic cell atlas of human psoriatic skin to date.

**Figure 1:**
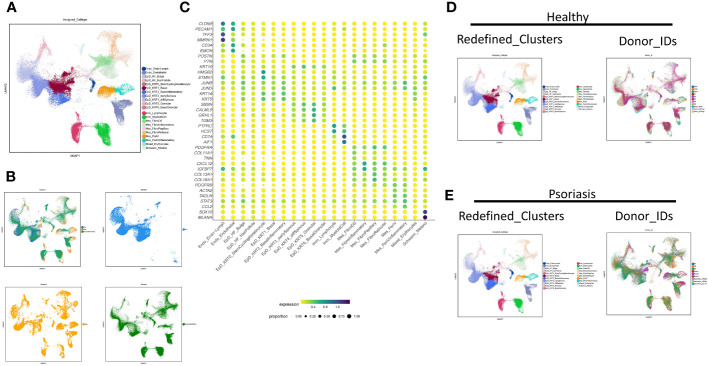
Neural-network learning based integrative analysis constructs the cell transcriptomic atlas of psoriatic skin. **(A, B)** UMAP visualization of the integrated cell transcriptomic atlas of human healthy and psoriatic skin. Each cell marked by “.”, colored by cluster names **(A)**. Original datasets are colored in an integrated visualization and separated visualization **(B)**. **(C)** Normalized gene expression defining each cell type of human healthy and psoriatic skin. Blue-to-red color gradient and dot size from small to large represent expression level and cell percent from low to high. **(D, E)** UMAP visualization of the integrated cell transcriptomic atlas of human healthy **(D)** and psoriatic skin **(E)**. Each cell marked by “.”, colored by cluster names at the left side. Each donor colored at the right side.

To make an insight further into the role of localized inflammation in resident skin cells during psoriasis, we performed an extensive clustering of epidermal and mesenchymal cells, revealing 21 main skin cell subtypes (**Figures 1A, B**). Employing interpretable learning, we recovered over 30,000 features of protein coding genes and lncRNAs, and assessed the marker genes for each cell type (**Figure 1C**). Various cell types and subtypes were defined by distinct markers. *CLDN5* and *PECAM1* distinguished two types of endothelial cells, with lymphatic endothelial cells (Endo_Endo-Lymph) highly expressing *TFF3* and *MMRN1*, and regular endothelial cells expressing *CD34* and *EMCN*. Nine epidermal cell subtypes were identified following skin cell zonation, and we observed a gradient change in marker genes (*KRT14*, *KRT5*, *SBSN*, *CALML5*, *GRHL1*, *TGM3*) from basal layers to granular layers, including basal cell (EpD_KRT1_Basal), basal-spinous intermedia cell (EpD_KRT3_earlySpinous), differentiating spinous-granular cell (EpD_KRT4_diffSpinous), granular cell (EpD_ERT5_Granular), and a few cells of basal-granular intermedia cell type (EpD_KRT6_BasalGranular). Inflammatory basal cell (EpD_KRT2_Basalinflammatory) were branched out from basal cell types due to the high expression of immune driver genes like *JUNB* and *JUND*. Our machine learning strategy also identified a small group of cells similar to hair follicle cells (EpD_HF_HairFollicle), but we could not identify any specific markers for this cell type due to the limited cell number.

We identified six mesenchymal cell subtypes, which could be classified into either *PDGFRA*-high/*PDGFRB*-low fibroblasts or *PDGFRB*-high/*PDGFRA*-low perivascular mural cells. Fibroblast cell types include fibroblast dermal papillae (Mes_FibroDS, labeled by *COL11A1*, *TNN*), inflammatory fibroblast (Mes_FibroInflammatory, labeled by *CXCL12*, *IGFBP7*), Papillary fibroblast (Mes_FibroPapillary, labeled by *COL13A1*-high/*COL18A1*-high), and Reticular fibroblast (Mes_FibroReticular, labeled by *COL13A1*-low/*COL18A1*-high). Perivascular mural cells include two main types: Mes_PeriV (*ACTA2*-high/*TAGLN*-high), and Mes_PeriVInflammatory (*STAT3*-high/*CCL2*-high). Immune cells comprised two main subtypes: lymphocytes (Imm_Lymphocyte, labeled by *PTPRC*-high/*HCST*-high) and myeloid cells (Imm_MyeloidCell, *CD74*-high/*AIF1*-high). Lastly, Schwann cells and melanocytes were grouped together as Schwann_Melano (*SOX10*, *MLANA*), and erythrocytes and a few undefined cells were categorized as Mixed_Erythrocytes with no typical maker genes.

The distribution of these cell subtypes was consistent across different datasets, indicating the reliability of the classification (**Figure 1B**). Furthermore, we visualized the cell clustering and individual distribution in both healthy and psoriatic skin, and the results were consistent with our clustering definitions (**Figures 1D, E**).

### The robustness of our current skin cell classification

**Figure 2:**
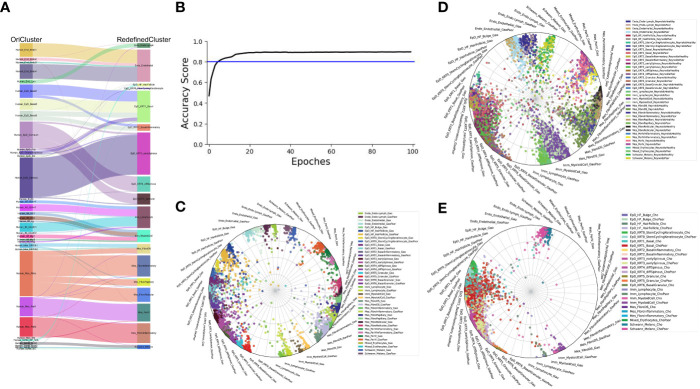
The robustness of our current skin cell classification. **(A)** Sankey plot visualization of the cell-type assignment between our original cell-types (left) and the currently redefined cell-types (right). Sankey flows between left and right are colored by the original cell-types. **(B)** Learning curve (Black) indicates the classification accuracy, blue line indication the threshold of 80% accuracy. **(C–E)** Radar plot visualization of the cell-type scores of skin cells from Gao dataset **(C)**, Reynold dataset **(D)** and Cheng/Cho dataset **(E)** in relation to the trained reference cell types (Gao dataset). Color coding based on cell types (left) as shown at the right side. The position of each dot indicates the cell-type score between that cell and the trained reference cell types, which are indicated outside each wheel bend.

Next, we examined the robustness of our redefined clusters. Firstly, we compared the redefined cell subgroups with the previous cell classifications of our published dataset (2). We found that the new epidermal and mesenchymal cell subtypes were more specific, while all cell subtypes remained consistent with the previous findings (**Figure 2A**). Furthermore, we employed the machine learning to evaluate the assignment accuracy under the condition of current cell-type classification and observed more than 90% accuracy in our defined cell-types under both healthy and psoriasis condition (**Figures 2B, C**). We used this ready-built learning model to predict the other two datasets involved in our study (**Figures 2D, E**), and all defined cell-types were correctly predicted except two cell-types (EpD_HF_HairFollicle and EpD_KRT6_BasalGranular) (7, 8). Both of these two cell-types are more specific to mouse hair skin, and rarely observed in human skin (data will be published separately). Interestingly, our redefined cell-types also correctly recognized the layer zonation that confirmed that Cho dataset (7) is only from human epidermis.

### The differential expression of lncRNAs in each cell-type between health and psoriatic skin

Through further analysis and experimental validation, our previous study uncovered that several subtypes of skin resident cells from epidermal and mesenchymal origins can participate in localized inflammation through alternative immune pathways and contribute to the development of psoriasis (2). To further study the differential expression and function of long non-coding RNAs in psoriasis at the single-cell level, we analyzed the expression of lncRNAs in all cell subgroups from our integrated psoriasis skin cell atlas and identified all lncRNAs that were specifically upregulated or downregulated in each skin cell type during the disease state. All lncRNAs identified are presented in **SI_Table 1**.

To expand upon the differential expression patterns of lncRNAs, we meticulously parsed our dataset to focus on individual cell subtypes of epidermal and mesenchymal cells, particularly under psoriasis conditions, as detailed in **SI_Table 1**. From this analysis, we have earmarked highly specific marker genes within the epidermal and mesenchymal cell clusters, setting them aside for deeper exploration in forthcoming experiments.

Through this analysis, we pinpointed pronounced fluctuations in the expression of *SNHG9* in an array of epidermal cell subtypes, including EpD_Basal2, EpD_Basal3, EpD_Corneum, EpD_Foli, EpD_Granular-spinous, EpD_Granular, and EpD_Spinous. In addition, our scrutiny unveiled distinct expression variations in *CALML3-AS1* within the EpD_Basal1 cell subtype, coupled with marked differentiation in the expression of *CARMN* in mesenchymal clusters, namely Mes_DP-DS1 and Mes_Fibro.

**Figure 3:**
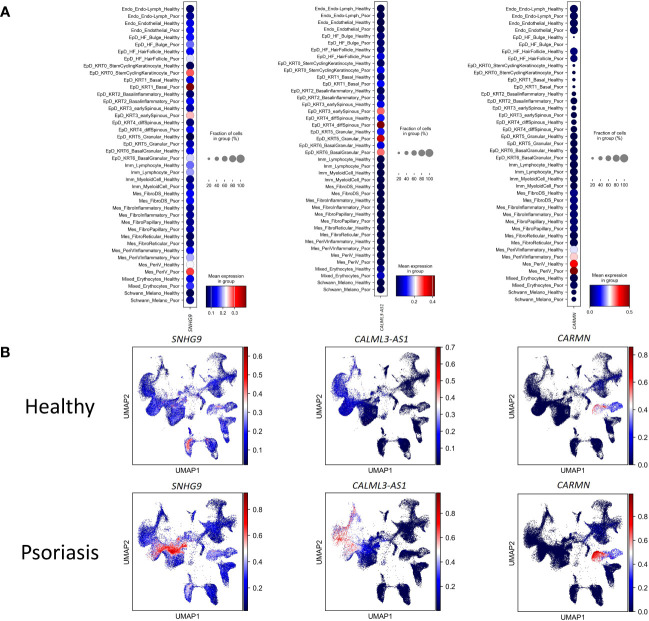
The differential expression of lncRNAs in each cell-type between health and psoriatic skin. **(A)** Normalized gene expression defining each cell type of human healthy and psoriatic skin. Blue-to-red color gradient and dot size from small to large represent expression level and cell percent from low to high. **(B)** UMAP visualization of comparing the lncRNAs’ expression between human healthy and psoriatic skin. LncRNA names are listed at the top of each sub plot. Blue-to-red color gradient represent expression level.

**Figure 4:**
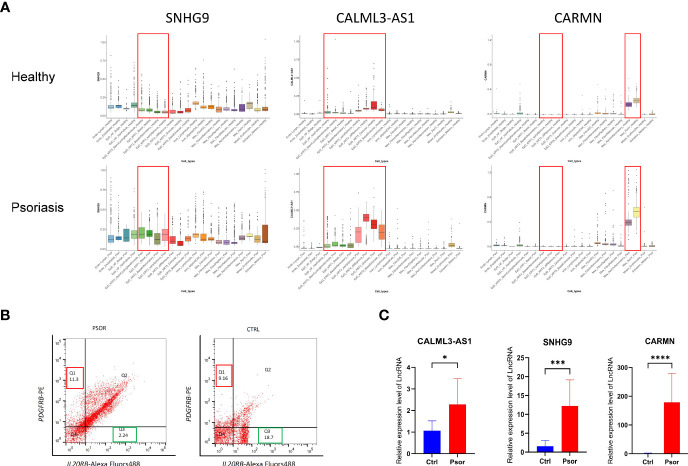
Experimental validation of the expression changes of lncRNAs *SNHG9, CALML3-AS1* and *CARMN* in psoriatic skin. **(A)** Box plot visualization of the expression change of lncRNAs *SNHG9, CALML3-AS1* and *CARMN* between health and psoriatic skin. lncRNA names are listed at the top of each plot, and the cell types with significantly altered lncRNA expression are highlighted with red box. **(B)** Representative images of flow cytometry sorting showing *PDGFRB*-positive mesenchymal cells (Q1 region) and *IL20RB*-positive epidermal cells (Q3 region) isolated from psoriasis and healthy control skin tissues. The numerical values within the boxes indicate the percentage of cells in each cell population (%). **(C)** Relative expression levels of lncRNAs *SNHG9, CALML3-AS1*, and *CARMN* in their respective cell types from psoriasis and healthy skin tissues. *P < 0.05, ***P < 0.001, ****P < 0.0001, t-test, n = 3 (mean ± SD).

To experimental validate the immunoregulatory potential of cell-type specific lncRNAs, we selected three lncRNAs (*SNHG9*, *CALML3-AS1*, and *CARMN*), and in silico examined their expression levels in different cell clusters under healthy and psoriasis conditions (**Figures 3A, B**). We found that *SNHG9* exhibited significantly higher expression in the EpD_KRT0_StemCyclingKeratinocyte, EpD_KRT1_Basal, EpD_KRT2_ Basalinflammatory and EpD_KRT3_earlySpinous clusters in psoriasis skin. *CALML3-AS1* showed increased expression in the EpD_HF_HairFollicle, EpD_KRT0_StemCyclingKeratinocyte, EpD_KRT1_Basal, EpD_KRT2_ Basalinflammatory, EpD_KRT3_earlySpinous, EpD_KRT4_diffSpinous, EpD_KRT5_Granular and EpD_KRT6_BasalGranular clusters in both normal and psoriasis skin, with significant upregulation observed in psoriasis. *CARMN* demonstrated elevated and significant expression in EpD_KRT2_ Basalinflammatory, EpD_KRT3_earlySpinous, EpD_KRT4_diffSpinous, Mes_PeriVInflammatory and Mes_PeriV clusters in both normal and psoriasis skin (**Figure 4A**). For detailed expression patterns of all lncRNAs in different cell clusters, please refer to **SI_Table 1**.

### Experimental validation of the expression change of lncRNAs *SNHG9*, *CALML3-AS1* and *CARMN* in psoriatic skin

To further confirm the expression patterns and potential functions of these lncRNAs in psoriasis and healthy skin cell subtypes, we performed the following experiments. First, we collected full-thickness skin tissue from patients with moderate to severe psoriasis and healthy volunteers, respectively, and obtained cell suspensions after digestion and dissociation. Then, we sorted the cells according to their cell type maker genes by flow cytometry (**Figure 4B**). *IL20RB* was used as a marker for epidermal cells, and *PDGFRB* was used as a marker for mesenchymal-derived cells. It has been previously validated that *IL20RB* and *PDGFRB* can be used as markers for epidermal and dermal mesenchymal cells, respectively, which is consistent with the single-cell RNA sequencing results in our experiment (13–15). We then used fluorescent quantitative PCR to detect the expression of specific lncRNAs in sorted epidermal and mesenchymal-derived cells. We found that in clinical skin samples, the expression levels of *SNHG9*, *CALML3-AS1* in epidermal cell types were significantly higher in psoriasis than in the healthy group. Similarly, the expression of *CARMN* in psoriatic mesenchymal-derived cell types was higher than in the healthy group. All results showed significant differences, as shown in the **Figure 4C**.

### Cell-specific lncRNAs *SNHG9*, *CALML3-AS1* and *CARMN* regulate localized inflammatory responses in skin resident cell clusters

Psoriasis is characterized by inflammation in the epidermis and dermis, and the regulation of localized inflammatory responses in epidermal and dermal cells by lncRNAs is of great importance for disease occurrence. EpD cells and Mes cells, as important resident cells in the epidermis and dermis, have been extensively studied and shown to play crucial roles in inflammation in psoriasis. Here, we further demonstrate the regulatory effects of *SNHG9*, *CALML3-AS1* and *CARMN* on the inflammatory responses of their respective cell types.

To further investigate the mechanisms of action of these cell subtype-specific expressed lncRNAs in psoriasis, we performed correlated genes analysis of *SNHG9*, *CALML3-AS1*, *CARMN* and GO enrichment analysis on the integrated single-cell data. We found that *SNHG9* and *CALML3-AS1* correspond to a high coexpression ratio with *NFKB1* and *STAT3* in the EpD cell clusters. *CARMN* correspond to a high co expression ratio with *NFLB1*, *STAT3*, *STAT5B*, and *STAT6* in Mes_PeriVInflammatory and Mes_PeriV clusters. The coexpression of lncRNAs and genes detail was showed in **SI_Table 2**. NF-kappa B (nuclear factor-kappa B) is a rapidly acting primary transcription factor found in all cell types. It is involved in cellular responses to stimuli such as cytokines and stress and plays a key role in regulating the immunological response to infections (16, 17). STATs (signal transducers and activators of transcription) are a family of seven transcription factors that form part of the JAK-STAT signaling cascade, which serves as the basis for the signal transduction mechanism of many cytokine receptors (18). STATs are activated by phosphorylation by JAKs. *STAT3*, in particular, has been implicated in several autoimmune diseases, including psoriasis (19, 20).

**Figure 5:**
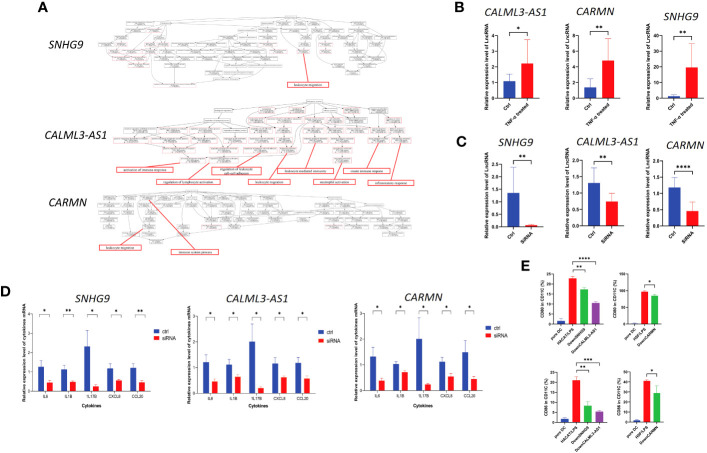
lncRNAs *SNHG9*, *CALML3-AS1*, and *CARMN* exhibit immune regulatory functions in skin-resident cell types. **(A)** GO enrichment analysis of *SNHG9*, *CALML3-AS1*, and *CARMN* on the integrated single-cell data. Immune-related functions are highlighted in red. **(B)** Relative expression levels of lncRNAs *SNHG9*, *CALML3-AS1* (in HACAT), and *CARMN* (in HSF) before and after treatment with TNF-α in cell lines. **(C)** Relative expression levels of lncRNAs *SNHG9*, *CALML3-AS1* (in HACAT), and *CARMN* (in HSF) before and after RNA interference in cell lines. **(D)** Relative expression levels of various cytokine mRNAs in cell lines from the TNF-α-treated group and the RNA interference group compared to the control group. **(E)** Flow cytometry analysis of *CD11C*-positive dendritic cells co-cultured with cell lines from the RNA interference group and the control group, showing the percentage of *CD80* and *CD86*-positive cells (%). *P < 0.05, **P < 0.01, ***P < 0.001, ****P < 0.0001, t-test, n = 3 (mean ± SD).

GO enrichment analysis revealed that these three lncRNAs are associated with biological processes related to excessive proliferation and inflammation observed in psoriasis skin in their respective cell clusters. The results of GO enrichment analysis are detailed in **SI_Tables 3**–**5**. Specifically, *SNHG9* in EpD cells is involved in leukocyte migration. *CALML3-AS1* in EpD cells functions in the activation of immune response, regulation of lymphocyte activation, regulation of leukocyte cell-cell adhesion, leukocyte migration, leukocyte mediated immunity, neutrophil activation, innate immune response, and inflammatory response. *CARMN* in Mes cell population is associated with lymphocyte migration and immune system process (**Figure 5A**). These biological processes indicate that *SNHG9*, *CALML3-AS1*, and *CARMN* have immune-related functions in their respective cell clusters. To further validate their roles in psoriasis, functional experiments were performed on these lncRNAs in cell lines.

Numerous studies have confirmed TNF-α as one of the key pathogenic cytokines in psoriasis and extensively used for inducing inflammatory conditions in epidermal cell lines (21–24). TNF-α can activate myeloid dendritic cells, induce epidermal proliferation and incomplete keratinization, and release host defense proteins and chemokines such as *CCL20* and *CXCL8*, leading to local skin thickening and inflammatory reactions that sustain the psoriatic phenotype (25–27). *IL1B* and *IL6* are potent pro-inflammatory cytokines that can induce Th1 generation of *IFNG* and synergistically promote inflammation and angiogenesis along with TNF-α (28–31). Our previous results have confirmed the involvement of *IL17B* in localized inflammation in psoriatic skin (2). Based on these experimental foundations, we treated HACAT and HSF cell lines with exogenous TNF-α to simulate the localized inflammatory response of epidermal and mesenchymal-derived cells in psoriasis, and analyzed the levels of *CXCL8*, *CCL20*, *IL1B*, *IL6*, and *IL17B* expression to assess the extent and status of inflammation.

We treated the immortalized human epidermal cell line HACAT and human dermal fibroblast cell line HSF with TNF-α and examined the expression levels of the aforementioned lncRNAs under inflammatory conditions. We found that after TNF-α treatment, the expression of *SNHG9*, *CALML3-AS1* in HACAT, and *CARMN* in HSF were all significantly upregulated compared to the control group, and all results showed statistical significance (*P* < 0.05) (**Figure 5B**). This finding is consistent with the expression results observed in the integrated atlas dataset of psoriatic skin and cell subtypes extracted from tissues, indicating elevated expression of *SNHG9*, *CALML3-AS1* and *CARMN* under localized inflammatory conditions in the skin.

Next, we further investigated the role of these lncRNAs in the TNF-α-induced inflammatory response in HACAT and HSF cell lines. RNA interference was used to knockdown the expression of *SNHG9*, *CALML3-AS1* in the epidermal HACAT cell line, and *CARMN* in the mesenchymal HSF cell line, respectively. qPCR analysis demonstrated that the expression of these lncRNAs in both the HACAT and HSF cell lines was significantly lower in the siRNA group compared to the control group, confirming successful interference, as shown in the **Figure 5C**.

Following TNF-α treatment of the successfully knocked-down lncRNA cell lines, we evaluated the mRNA expression levels of the inflammatory factors *CXCL8*, *CCL20*, *IL1B*, *IL6*, and *IL17B* using qPCR. We observed that, after lncRNA knockdown, the expression levels of TNF-α-induced *CXCL8*, *CCL20*, *IL1B*, *IL6*, and *IL17B* were all lower than those in the control group, and all results showed statistical significance (*P* < 0.05) (**Figure 5D**). Based on the above results, we can infer that these lncRNAs have the ability to regulate the localized inflammatory response in both epidermal and mesenchymal cell lines induced by TNF-α. Knocking down these lncRNAs leads to a reduction in the production of inflammatory factors induced by TNF-α.

### LncRNA *SNHG9*, *CALML3-AS1*, and *CARMN* can regulate the activation ability of corresponding skin cell types on dendritic cells

Dendritic cells (DCs) play a crucial role as upstream initiating cells in the localized inflammatory response in the skin, and their activation is essential for the pathogenesis of psoriasis, which is considered a key link in both the initiation and the maintenance phases of psoriasis (32). Based on previous sequencing results, we selected cell types expressing lncRNAs *SNHG9*, *CALML3-AS1*, and *CARMN*. After silencing lncRNA *SNHG9* and *CALML3-AS1* in the HACAT cell line, we co-cultured it with primary dendritic cells.

Here, we used *CD11C* as a marker to identify dendritic cells. *CD11C*, as a classical marker for dendritic cells, has been widely employed in research studies (33). The expression of *CD80*/*CD86* stimulatory molecules on dendritic cells can indicate their activation and their ability to initiate and regulate immune responses. We found that the levels of *CD80* and *CD86* expressed by dendritic cells induced by the epidermal cell line after lipopolysaccharide (LPS) 50ng/ml stimulation were regulated by *SNHG9* and *CALML3-AS1*, respectively. Specifically, interfering with the expression of *SNHG9* and *CALML3-AS1* in epidermal cells resulted in a decrease in the activation ratio of dendritic cells. We first used flow cytometry to measure the frequency of *CD80*/*CD86* in *CD11C*, which represents the activation level of dendritic cells, in primary dendritic cells before co-culture. We observed that untreated *CD11C*-positive dendritic cells exhibited a low proportion of *CD80*/*CD86*-positive cells, indicating that these dendritic cells were in an inactivated state.

To simulate the localized inflammation state in psoriasis, we treated HACAT and HSF cells with LPS respectively and then co-cultured them with dendritic cells. Flow cytometry was performed to assess the proportions of *CD80* and *CD86*-positive cells in *CD11C*-positive dendritic cells. This analysis allowed us to evaluate the activation level of dendritic cells and assess the extent of localized inflammation induced by skin-resident cells. Following siRNA-mediated knockdown of *SNHG9*, *CALML3-AS1* in HACAT cells, and *CARMN* in HSF cells, we repeated the aforementioned experiments. By measuring the activation level of dendritic cells, we analyzed the impact of *SNHG9*, *CALML3-AS1*, and *CARMN* on localized inflammation in skin cells. We observed a significant decrease in the proportion of *CD80* and *CD86*-positive cells in *CD11C*-positive dendritic cells upon knockdown of *SNHG9*, *CALML3-AS1* in HACAT cells, and *CARMN* in HSF cells, indicating an attenuation of dendritic cell activation (**Figure 5E**). These results suggest that lncRNAs *SNHG9*, *CALML3-AS1*, and *CARMN* in skin tissues can regulate the activation ability of respective cell types on dendritic cells. Interfering with the expression of these lncRNAs can inhibit the activation of dendritic cells, which may have potential regulatory effects on localized inflammation in psoriasis.

## Discussion

Psoriasis, characterized by excessive proliferation and abnormal differentiation of epidermal cells, involves a complex regulatory network with various cellular and molecular players. Even after the remission of clinical symptoms, psoriasis can relapse due to continued inflammatory reactions from certain skin-resident cells. This localized inflammation operates in tandem with known psoriasis immune pathways, causing a secondary immune response that prolongs skin inflammation (34). While biologics targeting IL-23, IL-17A, and TNF-α have shown effectiveness, discontinuation of treatment often leads to disease recurrence. This points towards the potential of localized skin inflammation, possibly fueled by epigenetic regulation within skin-resident cells, as an independent mechanism contributing to the persistence of the disease.

The advent of scRNAseq technology allows the uncovering of rare cell subpopulations playing significant roles in psoriasis, extending our understanding beyond the traditional classification boundaries of the epidermis and dermis (35–39). Thanks to scRNAseq, a variety of single-cell atlases such as the Human Cell Atlas and Tabula Muris have been established. These atlases, comprising samples from different tissues, laboratories, and experimental conditions, inevitably carry batch effects. Therefore, the development of integration methods to overcome these batch effects has become a priority in recent years. By meticulously merging 106,675 cells from healthy human skin and 79,887 cells from psoriatic human skin, we have crafted the most detailed and expansive transcriptomic cell atlas of human psoriatic skin to date, distinctly surpassing the boundaries set by previous datasets. The core strength of our study lies in the sophisticated integration of a sizable cell atlas facilitated by our neural-network learning pipeline. This integrated approach mitigates the batch effects that have been a persistent challenge in other datasets and enhances the accuracy and depth of skin cell classification, thus uncovering the underlying mechanisms of psoriatic skin pathogenesis. Furthermore, our pioneering approach in employing interpretable learning enabled us to mine over 30,000 features of protein-coding genes and lncRNAs, setting a new benchmark in the depth and breadth of cellular analysis, distinguishing our dataset as a markedly superior tool in the quest to unravel the mysteries of psoriasis.

Our approach has spotlighted a rich diversity of skin cell subtypes, accentuating the finer nuances that govern the complex dynamics of psoriasis. The robustness and specificity in our newly defined epidermal and mesenchymal cell subtypes are a testament to our integrated dataset’s heightened accuracy and reliability, distinctly setting it apart from earlier studies. Notably, our methodology demonstrated an accuracy exceeding 90%, showcasing our significant advancements in cell classification and underscoring the potential to venture deeper into uncharted territories of cellular dynamics in psoriasis.

Beyond identifying cellular subtypes, our study has honed in on the critical role of long non-coding RNAs (lncRNAs) in modulating localized inflammation in psoriasis. This focus, previously underexplored, now opens up a new frontier for research and therapeutic development, underscoring the advantage of our integrated approach in spotlighting promising candidates for further investigation.

As we advance, we remain responsible to unravel the regulatory mechanisms underlying psoriasis pathogenesis. Our consolidated dataset is a novel and powerful resource that promises to catalyze the next wave of breakthroughs in this field. Moreover, the public online accessibility of our dataset ensures that it serves as a collaborative platform, promoting further innovation and discoveries in psoriasis research.

In our previous report, we demonstrated that local resident cells, including epidermal cells and mesenchymal cells, display an immune-priming profile that amplifies inflammation in psoriatic skin (2). As a key player in this network, epidermal cells are impacted by multiple factors including genetics, cytokines, receptors, metabolism, cell signaling pathways, transcription factors, non-coding RNAs, antimicrobial peptides, and diverse functional proteins (40). These factors collectively contribute to the onset and progression of psoriasis. In concert with mesenchymal-derived fibroblasts and endothelial cells, Epidermal cells instigate tissue remodeling through endothelial cell activation and proliferation, as well as extracellular matrix deposition (41, 42). Recent studies have also found that inhibitors of glucose transporter 1 (Glut1), pyruvate kinase M2 (PKM2), and 2-deoxy-D-glucose (2DG) can alleviate the severity of psoriasis-like skin inflammation (43–45).

Mesenchymal cells are widely present in connective tissues, as well as in the skin and subcutaneous tissues, including dermal fibroblasts and pericytes in the dermis, and adipocytes in subcutaneous tissues (46, 47). Dermal fibroblasts, once activated, have the potential to play an important role in the development of psoriasis. They can be recruited to skin tissue that is damaged, inflamed, or healing, and they can activate immune cells and modulate inflammation levels. In addition, activated fibroblasts are capable of secreting cytokines, leading to excessive proliferation of keratinocytes, a crucial factor in the progression of psoriasis (46, 48).

Furthermore, immune cells, especially dendritic cells, play pivotal roles in both the initiation and maintenance phases of psoriasis (32, 41). DCs, activated by epidermal cells, release inflammatory cytokines such as TNF-α, IL-12, and IL-23, which further activate Th1 and Th17 cells. These immune cells then secrete additional inflammatory cytokines, forming a positive feedback loop that exacerbates psoriasis-associated inflammation and epidermal hyperproliferation (40, 49).

Emerging evidence points towards long non-coding RNAs (lncRNAs) as critical epigenetic regulators in psoriasis (49–51). Through high-throughput sequencing, numerous differentially expressed lncRNAs have been identified in psoriasis skin tissues, indicating their crucial roles in psoriasis pathogenesis (52–57). Yet, previous RNA sequencing largely focused on cell types with the largest changes in psoriasis, leading to an underrepresentation of other important cell subtypes.

Through this integrated dataset, we identify differentially expressed lncRNAs in cell subtypes implicated in psoriasis and experimentally validate the functions of lncRNAs *SNHG9*, *CALML3-AS1* in epidermal cells, and *CARMN* in mesenchymal cells. At present, study on these lncRNAs is very limited, and the mechanism by which they participate in regulating immune function is still unclear. *SNHG9*, affiliated with the lncRNA class, is an RNA gene that is currently believed to promote the proliferation of various types of tumor cells through multiple pathways, including phosphatidylinositol binding, inhibition of autophagy, and participation in methylation regulation (58–60). *SNHG9* secreted by adipocyte-derived exosomes can alleviate inflammation and endothelial cell apoptosis by inhibiting *TRADD* expression (61). However, it is unclear whether this anti-endothelial cell apoptosis property is related to the microvascular endothelial proliferation observed in psoriasis. Existing research on the *CALML3-AS1* is relatively sparse, and the principal focus of existing studies is cancer biology. Current hypotheses suggest that *CALML3-AS1* may exert a regulatory influence on tumorigenesis via mechanisms that may include functioning as a molecular sponge for microRNAs or operating as a transcriptional regulator (62). *CARMN* plays a role in smooth muscle-related diseases and tumors. *CARMN* can maintain a contractile phenotype by binding to myosin (17). *CARMN* in smooth muscle cells regulates cell plasticity and atherosclerosis by interacting with serum response factors (63), and its deficiency can accelerate atherosclerosis progression (64). We sorted epidermal and mesenchymal cell types from the skin tissues of patients with psoriasis and healthy volunteers, and verified that these lncRNAs were significantly increased in the corresponding cell types under disease conditions compared to healthy controls. They have been confirmed to have immune function through *in vitro* cell experiments, silencing these lncRNAs in skin resident cell clusters can attenuate the expression of inflammatory cytokines and inhibit dendritic cell activation under inflammatory conditions, suggesting a potential therapeutic strategy to reduce localized inflammation in psoriasis. This research provides valuable insights into the development of psoriasis at the single-cell level and indicates potential targets for therapeutic interventions.

## Data availability statement

The original contributions presented in the study are included in the article/**Supplementary Material**. Further inquiries can be directed to the corresponding authors.

## Ethics statement

The studies involving humans were approved by The Second Affiliated Hospital of Harbin Medical University. The studies were conducted in accordance with the local legislation and institutional requirements. The participants provided their written informed consent to participate in this study.

## Author contributions

YG: Data curation, Methodology, Validation, Writing – original draft, Writing – review & editing. MN: Investigation, Methodology, Project administration, Writing – original draft. XY: Methodology, Investigation, Writing – original draft. CL: Methodology, Writing – original draft, Formal Analysis, Project administration. LL: Methodology, Project administration, Resources, Writing – original draft. GY: Formal Analysis, Project administration, Resources, Writing – original draft. YL: Conceptualization, Funding acquisition, Writing – review & editing. YH: Conceptualization, Investigation, Writing – review & editing.
